# How to Examine the Mouth of an Infant

**Published:** 1901-11-30

**Authors:** 


### HOW TO EXAMINE THE MOUTH OF AN INFANT.

Dr. Jacob Sobel[1] says that for a thorough inspection of the mouth in infants and children three conditions are absolutely necessary, namely, good position, good light, and a good tongue depressor. A great deal of unnecessary exertion is often wasted in attempts to "hold the child down" in unnatural positions. The ideal position is to have the back of the child rest against the mother's right breast, its head against her shoulder, her left hand holding the child's knees, her right hand its hands. The left index finger and thumb of the physician are placed on the infant's temporal region, and with the palm the head is held firmly against the mother's shoulder. Without good light nothing can be accomplished. Direct daylight, not sunlight, answers every purpose. Or a forehead reflector may be used. At night the forehead mirror should always be used, unless one uses a specially-constructed pocket electric lamp. In making a systematic examination, the depressor is first inserted at the left angle of the mouth, the cheek and lips everted; then at the right angle and the cheek and lips everted at that side also. The spatula then catches the frenum and the under surface of the tongue, and thus the frenum and the floor of the mouth are observed; the spatula being removed, the upper surface of the tongue is then viewed. The tongue is then firmly depressed, and the hard and soft palates, fauces, tonsils, pharynx, and in the vast majority of cases the epiglottis are observed.

[1] New York Medical News, Nov. 2.

